# Multimodal Imaging in a Case of Localized Suprachoroidal Hemorrhage

**DOI:** 10.18502/jovr.v15i1.5956

**Published:** 2020-02-02

**Authors:** Avadhesh Oli, Divya Balakrishnan

**Affiliations:** ^1^Smt Kanuri Santhamma Center for Vitreo Retina Services, L. V. Prasad Eye Institute, Hyderabad, India

**Keywords:** Choroidal Granuloma, Choroidal Mass, Melanoma, Supra Choroidal Hemorrhage

## Abstract

**Purpose:**

To report a case of localized suprachoroidal hemorrhage presenting as a choroidal mass.

**Case Report:**

A 66-year-old woman presented with sudden onset pain in the right eye, one week following uneventful cataract surgery. The best corrected visual acuity (BCVA) was 20/160 and fundus examination showed a brown elevated choroidal mass temporal to the fovea in the right eye with normal retina and retinal vessels over it. The differential diagnoses considered were choroidal granuloma, melanoma, choroidal hemangioma, posterior scleritis, and localized suprachoroidal hemorrhage (SCH). Fluorescein angiography (FA) and indocyanine green (ICG) angiography were unremarkable except for mild disc leakage; B-scan showed a choroidal mass with high surface reflectivity and low internal reflectivity, and optical coherence tomography (OCT) showed an elevation of retino-choroidal complex with hyporeflective mass in the outer choroid with choroidal folds suggestive of SCH. Her systemic evaluation showed raised erythrocyte sedimentation rate (ESR) and consolidation in the upper lobe of the right lung. The patient did not take any additional treatment for her eye and the lesion regressed and visual acuity improved to 20/30 in one month.

**Conclusion:**

Delayed spontaneous suprachoroidal hemorrhage can present as a choroidal mass. Multimodal imaging helps to differentiate it from other sight-threatening and life-threatening ocular diseases.

##  INTRODUCTION

Spontaneous suprachoroidal hemorrhage (SCH) is a rare occurrence associated with intraocular surgeries. A few cases of SCH following Valsalva maneuver and delayed postoperative hemorrhage have been reported.^[1]^ A localized spontaneous SCH can cause diagnostic dilemma by presenting as a choroidal mass. Multimodal imaging including fluorescein angiography (FA), indocyanine green angiography (ICG), ultrasound B scan, and optical coherence tomography (OCT) help to differentiate various choroidal mass lesions like melanoma, choroidal granuloma, choroidal hemangioma, posterior scleritis, and suprachoroidal hemorrhage (SCH). The present case highlights the role of multimodal imaging in differentiating SCH from other choroidal masses.

##  CASE REPORT

**Figure 1:**
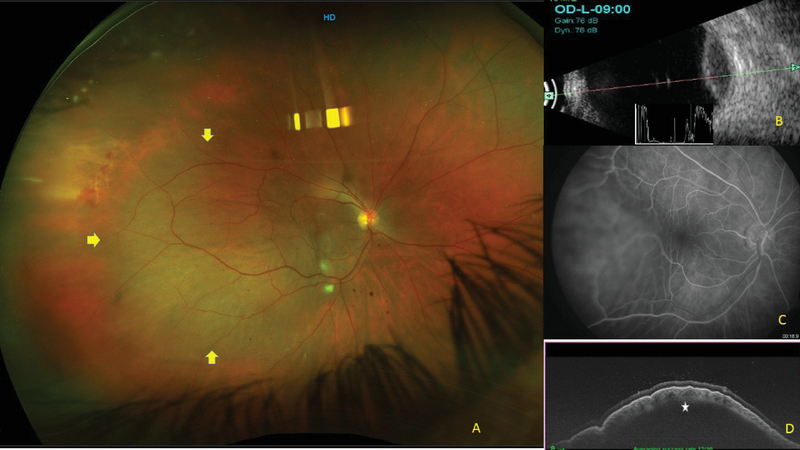
(A) Fundus picture: Yellow arrows show the extent of the lesion. (B) B scan shows a well-defined mass with high surface reflectivity and low internal reflectivity. No sub-Tenon's fluid. (C) FA and ICG show normal fluorescence and late disc leakage. (D) OCT shows elevation of the retino-choroidal complex with hyporeflective lesion in outer choroid with smooth anterior surface and a vertical scan showing choroidal folds.

**Figure 2:**
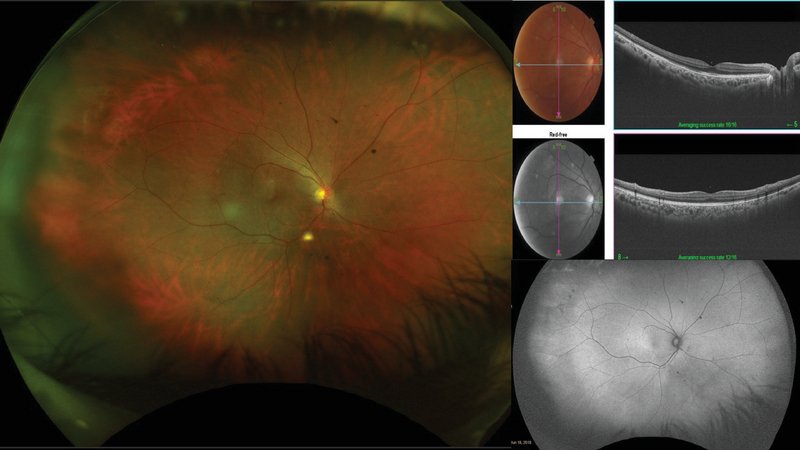
Fundus photo and OCT showing complete resolution of mass.

A 66-year-old woman presented with a history of sudden onset of excruciating pain in her right eye. She had undergone phacoemulsification and posterior chamber intraocular lens (PCIOL) implantation in the right eye a week before. Her systemic history was otherwise normal except for osteoarthritis for which she occasionally used analgesics. On examination, her visual acuity was 20/160 and 20/60 in right and left eyes, respectively. Slit lamp examination showed few Descemet's folds and PCIOL in the right eye and cataract in the left eye. Intraocular pressure (IOP) was 14 and 16 mm Hg by applanation tonometry. Fundus showed a localized dome-shaped elevated choroidal mass temporal to the fovea with no orange pigment changes or subretinal fluid, with a diameter of about 5–6 disc diameters (DD), with overlying choroidal folds, and the presence of a few retinal hemorrhages in the temporal periphery in the right eye [Figure 1 (A)]. The retina of the left eye was normal. The patient was treated with topical steroids (prednisolone acetate eye drops 1% six times a day) and oral paracetamol 650 mg thrice a day. Keeping a differential diagnosis of melanoma, choroidal granuloma, choroidal hemangioma and posterior scleritis in mind, the patient was referred to the oncology clinic. The patient underwent multimodal imaging and systemic workup. FA showed normal fluorescence pattern, except for minimal disc leakage and overlying choroidal folds in the right eye [Figures 2 (A–C)]. ICG showed normal fluorescence pattern [Figures 2 (D–E)]. The fundus autofluorescence was normal in both eyes. Ultrasound B scan showed a well-defined mass with high surface reflectivity and low to medium internal reflectivity without sub-Tenon's fluid [Figure 1 (B)]. OCT showed an elevation of retinochoroidal complex over a hyporeflective space in the outer choroid with a smooth anterior surface, pushing the inner choroid, along with minimal subretinal fluid [Figures 1 (C, D)]. The absence of intrinsic circulation and a normal FA and ICG ruled out choroidal melanoma and hemangioma. Systemic workup showed a positive Mantoux test (24 mm), and the chest X-ray showed lung consolidation in the upper lobe. The differential diagnoses considered were of either SCH or choroidal granuloma. She was referred to an internist to start antitubercular therapy (ATT). The patient came for a review at one month and had not taken any treatment for her eye other than topical steroids and paracetamol tablet. The lesion had spontaneously regressed and visual acuity had improved to 20/30 with a normal OCT [Figure 2].

**Table 1:** Key clinical and multimodal imaging features of differentials of choroidal mass


**Features **	**Melanoma**	**Granuloma**	**Suprachoroidal Hemorrhage**	**Hemangioma**	**Posterior scleritis**
**Clinical features**					
Pain	No	Rare	Yes	No	Yes
Choroidal folds	May be seen at the edges	Rare	Yes	No	Yes
B scan	Low	Low	Echolucent	High	Low, T sign
Internal reflectivity								
FFA ICG	Double circulation	Early hypo fluorescence, late hyper fluorescence Persistent hypo fluorescence	No remarkable features	Early bright hyper fluorescence, late diffuse hyper fluorescence Early hyper fluorescence, late washout	Pinpoint leaks Zonal hyper with pinpoint leak
OCT	Dome-shaped mass, subretinal fluid, Shaggy photoreceptors	Hypo reflective thickening of the choroid, the outer boundary of choroid can be seen	Hypo reflective lesion in outer choroid with elevated retinochoroidal complex, choroidal folds	Elevated smooth choroidal mass, rounding of choriocapillaris	Increased choroidal thickness with retinal folds
	
	
FFA, fundus fluorescein angiography; ICG, indocyanine green angiography; OCT, optical coherence tomography					

In retrospect, a choroidal mass with no changes on FA or ICG, which was dome shaped, optically lucent in OCT and B scan, showing high surface reflectivity with low internal reflectivity and a spontaneous resolution were suggestive of a localized SCH. The typical FA and ICG features of choroidal granuloma^[2]^ (early hypo and late hyper-fluorescence in FA and persistent hypo-fluorescence in ICG) were not seen in this patient, but the disc showed minimal leakage in the late phase.

##  DISCUSSION

Spontaneous suprachoroidal hemorrhage is a relatively rare differential diagnosis of a choroidal mass lesion, which accounts for 2% of pseudo melanomas.^[3]^ The important risk factors are old age, hypertension, Valsalva maneuver and use of anti-coagulants. The dome-shaped elevation in the fundus due to SCH is usually limited to the area of fibrous adhesions concurrent with vortex veins; however, massive SCH can extend posteriorly.^[4]^ As it closely mimics other life-threatening conditions like choroidal melanoma, metastasis, and granuloma, patients are usually investigated.^[2,3]^

Though cataract surgery was uneventful in our patient, it could have been the precipitating factor.^[5]^ The patient not only presented with ocular pain, which is usually seen associated with posterior scleritis or granuloma, but also with features suggestive of SCH, which caused a diagnostic dilemma.^[1]^ The fundus lesion of a brown elevated mass mimicked melanoma, hemangioma, and granuloma, but the features of melanoma-like orange pigments were absent.

Multimodal imaging helps to differentiate various choroidal mass lesions^[6]^ [Table 1].

The B scan showed acoustic hollowing, but no choroidal excavation. This feature of SCH has already been described.^[1,6]^ The presence of choroidal folds over the lesion with background normal fluorescence in FA and choroidal folds over an optically empty elevated lesion in OCT is a feature of SCH.^[6,7]^ A choroidal hemangioma would show high surface and internal reflectivity in B scan and bright hyperfluorescence on FFA. The absence of typical features of granuloma in FA and OCT ruled out granuloma, but the disc leakage in the late phase of FA along with a history of pain created a diagnostic dilemma with granuloma and posterior scleritis. The disc leakage in this case, could be explained by postoperative inflammation.

A cautious interpretation of lab tests like Mantoux and meticulous analysis of multimodal imaging in correlation with clinical features is required to make the correct diagnosis of cases such as this one. Patients may be closely observed for one or two months for spontaneous resolution in case of strong clinical suspicion of localized SCH, and invasive investigations and extensive systemic workup may be considered in cases of non-resolution.^[6]^

Even though localized SCH is a rare entity and regresses spontaneously, it is rarely thought of by ophthalmologists in the differential diagnosis of a choroidal mass.^[7]^

##  Financial Support and Sponsorship

Nil.

##  Conflicts of Interest

There are no conflicts of interest.
